# Torsion of Wandering Gallbladder following Colonoscopy

**DOI:** 10.1155/2013/808751

**Published:** 2013-07-17

**Authors:** Sean R. Warfe, Hannah Dobson, Matthew K. H. Hong, Weranja K. B. Ranasinghe, Peter R. Thomas, Adam G. Cichowitz

**Affiliations:** Department of Surgery, Northeast Health Wangaratta, Wangaratta, VIC 3677, Australia

## Abstract

Torsion of the gallbladder is an uncommon condition that may present as an acute abdomen. Its preoperative diagnosis can often be challenging due to its variable presentation, with specific sonographic signs seen infrequently. We describe, to our knowledge, the first case of torsion of a wandering gallbladder following a colonoscopy in a 69-year-old female who presented with acute abdominal pain after procedure. This was discovered intraoperatively, and after a subsequent cholecystectomy, she had an uncomplicated recovery.

## 1. Introduction

Torsion of the gallbladder is an uncommon condition that may present as an acute abdomen. Its preoperative diagnosis can often be challenging due to its variable presentation, with specific sonographic signs seen infrequently. We present the case of a 69-year-old female who presented with an acute abdomen, three hours after colonoscopy. To our best knowledge this is the first published case of torsion of a gallbladder caused by colonoscopy.

## 2. Case Study

A 69-year-old female presented to our surgical unit with acute onset epigastric pain and nausea three hours after an elective colonoscopy. The colonoscopy was performed to investigate altered bowel habit and left iliac fossa pain. No cause for the pain was demonstrated, but a long redundant, tortuous, and highly spastic colon with some haemorrhoids was noted. Physical examination revealed mild tenderness in the right upper quadrant. Initial investigations included normal liver function tests and inflammatory markers. A chest radiograph did not demonstrate any free gas under the diaphragm. 

**Figure 1:**
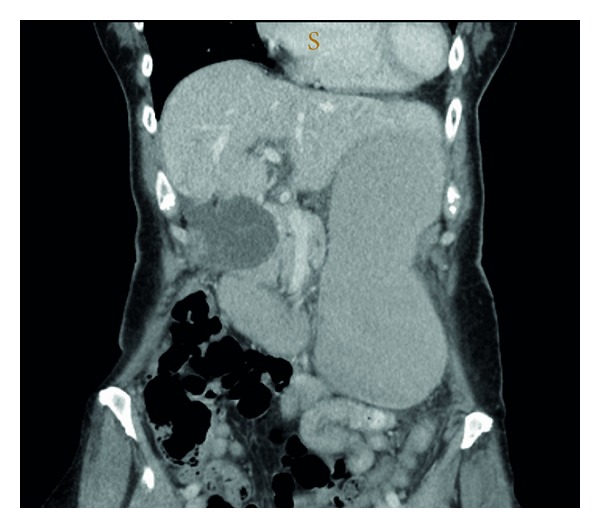
Reconstructed coronal images on computed tomography demonstrating the dilated and relatively inferiorly placed gallbladder. Acute gastric distension was also present due to duodenal compression by the very dilated gallbladder (not shown).

The patient's pain worsened markedly over a 12-hour period of observation. Computerized tomography (CT) of the abdomen was obtained which showed a grossly distended gallbladder and stomach with hepatic duct dilatation, but no cholelithiasis (Figure 1). Transabdominal ultrasonography demonstrated thickening of the gallbladder wall of 8 mm, but again no gallstones were evident. 

Repeat liver function tests demonstrated a new cholestatic picture (bilirubin 49 (NR = 0–20) *μ*mol/L, alanine transaminase 68 (NR = 0–55) units/L, alkaline phosphatase 76 (NR = 35–110) units/L, and gamma-glutamyl transferase 47 (NR = 0–50) units/L) and serum C-reactive protein was elevated to 177 mg/L. A presumptive diagnosis of acute acalculous cholecystitis was made, and the patient proceeded to theatre for a laparoscopic cholecystectomy. 

**Figure 2:**
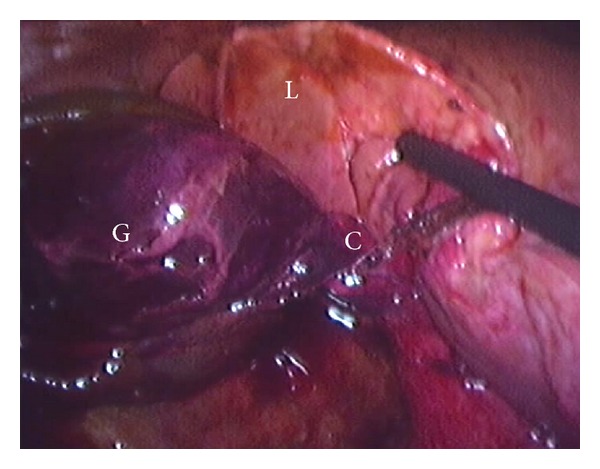
Intraoperative view of gangrenous wandering gallbladder (G) completely separate from the liver (L) and torted clockwise around the cystic duct and mesentery (C).

At laparoscopy, a gangrenous hypermobile gallbladder was seen. This was found to be completely free from the liver and connected to the biliary tree by a cystic duct within a narrow mesentery around which it had undergone 360° of torsion (Figure 2). The gallbladder was necrotic and haemorrhagic. 

Intraoperative cholangiography demonstrated dilated intrahepatic ducts and common hepatic duct down to the level of the cystic duct and gallbladder. Cholecstectomy was performed without difficulty after the cystic duct and vessels were isolated, clipped, and divided. 

Histopathological examination of the specimen confirmed acute gangrenous cholecystitis and the absence of cholelithiasis. The patient had an uncomplicated recovery and was discharged after two days. 

## 3. Discussion

Over 300 cases of gallbladder torsion have been reported in the literature since its initial description in 1898 [1]. The median age of onset is 77 years, and the patient is typically female. Most reported cases of torsion involve a floating gallbladder, which is attached to the liver by a mesentery [2], but the present case involved a so-called wandering gallbladder. This anatomic variant leads to hypermobility of the fundus and body of the gallbladder, which is attached to the biliary system solely by the cystic duct and mesentery with no attachment to the liver, rendering it prone to torsion [3].

The earliest description of torsion of a wandering gallbladder was by Ziegler in 1952 [4], and fewer than 10 cases of this unusual anatomy have been reported [5]. Preoperative diagnosis of torsion of a wandering gallbladder is challenging as the clinical presentation can be variable with recurrent episodes of nonspecific abdominal pain, presumably due to intermittent torsion [6]. Patients presenting with a persistent acute abdomen after a colonoscopy will always require imaging, such as upright chest X-ray, to rule out perforation. If this demonstrates no signs of perforation such as air under the diaphragm, further imaging such as CT or ultrasound should be considered. Triple-contrast or double-contrast CT can be used in those where perforation is still suspected. Specific ultrasonographic signs including rotation on its principal axis and pericholecystic oedema are rarely present [3]. A recent systematic review suggests that the rate of diagnosis with ultrasound has increased to approximately 26% [1]. In the absence of radiological findings of torsion with focal abdominal pain and tenderness in the right upper quadrant, laparoscopy should be considered in order to establish a definitive diagnosis with the view to performing a cholecystectomy if torsion is present.

To our knowledge, this is the first reported case of wandering gallbladder torsion occurring in the context of colonoscopy. Although causality between colonoscopy and gallbladder torsion cannot be proven, the development of acute right-upper-quadrant pain just hours after colonoscopy leads us to postulate that the colonoscope mechanically rotated the gallbladder during the procedure, which lead to the torsion of the wandering gallbladder. It is also conceivable that gas insufflation of the colon may have resulted in torsion of a hypermobile gallbladder. Interestingly, the colonoscopy was originally performed to investigate long-standing intermittent abdominal pain, perhaps due to intermittent incomplete torsion of the wandering gallbladder. 

## 4. Conclusion

In conclusion, torsion of the gallbladder is a rare but important cause of the acute abdomen. Preoperative identification of gallbladder torsion is challenging, but specific ultrasound findings can be used to assist with diagnosis. Management of the condition is relatively easy with laparoscopic cholecystectomy.

## References

[1] Reilly DJ, Kalogeropoulos G, Thiruchelvam D (2012). Torsion of the gallbladder: a systematic review. *HPB*.

[2] Boonstra EA, van Etten B, Prins TR, Sieders E, van Leeuwen BL (2012). Torsion of the gallbladder. *Journal of Gastrointestinal Surgery*.

[3] Dorland N (2003). *Dorland's Illustrated Medical Dictionaryed*.

[4] Ziegler H (1952). On the genesis of the so-called wandering gall-bladder, explained in a case of pedicle torsion. *Wiener medizinische Wochenschrift*.

[5] Morales AM, Tyroch AH (2008). Wandering gallbladder. *American Journal of Surgery*.

[6] Chiavarini RL, Chang SF, Westerfield JD (1975). The wandering gallbladder. *Radiology*.

